# Tribological Properties of Diamond/Diamond-like Carbon (DLC) Composite Coating in a Dry Environment

**DOI:** 10.3390/ma18163879

**Published:** 2025-08-19

**Authors:** Chengye Yang, Zhengxiong Ou, Yuanyuan Mu, Xingqiao Chen, Shihao Yang, Peng Guo, Nan Jiang, Kazuhito Nishimura, Xinbiao Mao, Hui Song, He Li

**Affiliations:** 1 Department of Applied Chemistry, Petroleum and Chemical Industry Key Laboratory of Organic Electrochemical Synthesis, State Key Laboratory of Green Chemistry Synthesis Technology, Zhejiang University of Technology, Hangzhou 310014, China; yangchengye@nimte.ac.cn; 2 State Key Laboratory of Advanced Marine Materials, Ningbo Institute of Materials Technology and Engineering, Chinese Academy of Sciences, Ningbo 315201, China; ouzhengxiong@nimte.ac.cn (Z.O.); muyuanyuan@nimte.ac.cn (Y.M.); chenxingqiao@nimte.ac.cn (X.C.); yangshihao@nimte.ac.cn (S.Y.); guopeng@nimte.ac.cn (P.G.); jiangnan@nimte.ac.cn (N.J.); kazuhitonishimura@nimte.ac.cn (K.N.); 3 Center of Materials Science and Optoelectronics Engineering, University of Chinese Academy of Sciences, Beijing 100049, China

**Keywords:** diamond, DLC, composite coating, dry friction, mechanism

## Abstract

In this study, a diamond/diamond-like carbon (DLC) composite coating was designed and fabricated utilizing a combination of chemical vapor deposition (CVD) and magnetron-sputtering-assisted ion beam deposition. This was designed to cope with severe problems such as high wear due to insufficient lubrication under dry sliding conditions with a single diamond. The tribological properties of the fabricated coatings under dry conditions were comparatively evaluated. The results demonstrate that the diamond/DLC composite coatings significantly enhance the tribological performance relative to their single-layer diamond counterparts. Specifically, a 33.73% reduction in the average friction coefficient and a 39.55% decrease in the average wear rate were observed with the MCD (microcrystalline diamond/DLC coating. Similarly, a 16.85% reduction in the average friction coefficient and a 9.69% decrease in the average wear rate were observed with the UNCD (ultrananocrystalline diamond)/DLC coating. Analysis of the worn track morphology and structure elucidated the underlying friction mechanism. It is proposed that the DLC top layer reduces the surface roughness of the underlying diamond coating and mitigates abrasive wear in the dry environment. Furthermore, the presence of the DLC film promotes graphitization via phase transition during sliding, which enhances lubricity and facilitates the establishment of a smooth friction interface.

## 1. Introduction

Diamond possesses a cubic crystal structure in which each carbon atom forms covalent bonds with four neighboring carbon atoms via sp^3^ hybridization, resulting in a regular tetrahedral arrangement [1]. The diamond coatings, recognized as being excellent solid lubricants, have garnered significant attention owing to their exceptional properties, including their ultra-high hardness (50–100 GPa), outstanding thermal conductivity (~2100 W/m·K) [2], low friction coefficient (0.05–0.15), and excellent chemical stability [3]. Based on the grain size, polycrystalline diamond coatings are typically categorized into three types: microcrystalline diamond (MCD), nanocrystalline diamond (NCD), and ultrananocrystalline diamond (UNCD) [4,5,6]. In practical applications, the relatively large grain size of MCD coatings often leads to increased surface roughness, which detrimentally elevates the interfacial friction coefficient. Conversely, the synthesis of NCD or UNCD coatings is frequently accompanied by the formation of non-diamond carbon phases (e.g., sp^2^-bonded carbon). While this can contribute to a lower friction coefficient, it often concurrently reduces the coating’s adhesion strength. However, to meet the demands of increasingly severe service environments for modern equipment, the development of diamond coatings exhibiting a simultaneously low surface roughness, low friction coefficient, and high adhesion strength has become critically important. As a critical technology for extending component service life, the effective application of wear-resistant coatings necessitates precise tailoring to specific operating conditions [7]. The selection and optimization of coating solutions require systematic consideration of multiple factors, including substrate properties, the service environment [8,9] (e.g., temperature, chemical media, atmosphere), dominant wear mechanisms (such as abrasive, adhesive, erosive, and corrosive wear, including their synergistic interactions), and the overall performance requirements [10] (e.g., hardness, bond strength, corrosion resistance, thickness, coefficient of friction, surface roughness, dimensional accuracy). Furthermore, distinct application scenarios—such as aero-engine high-temperature components, hydraulic rods, mining machinery, precision molds, or food processing equipment—impose highly specific and often stringent performance demands. Process feasibility, cost-effectiveness, and adherence to industry regulations are also critical considerations. Consequently, a comprehensive understanding of these multifaceted parameters and application-specific requirements forms an essential prerequisite for the effective and reliable implementation of wear-resistant coatings.

Previous studies have demonstrated that MCD coatings typically exhibit a higher hardness and adhesion strength compared to their nanocrystalline counterparts. Research on the tribological properties of diamond coatings has primarily focused on elucidating friction mechanisms under diverse conditions and optimizing the crystal structure [11,12,13,14]. For instance, Xu et al. [15] reported that NCD films maintain the ultra-long service life and exceptional wear resistance in high-radiation environments. They attributed this performance to the formation of reliable, amorphous-structured tribofilms on counterpart balls, providing superior lubrication. Ramasubramanian K et al. [16] compared MCD coatings on reaction-bonded silicon carbide substrates in dry and seawater environments. The superior friction performance in seawater was linked to a lubricating film at the sliding interface. Wear-track analysis confirmed the presence of this layer, composed of carbon, silicon, and oxygen, with friction reduction primarily being attributed to the silica layer formation. Hiroyuki Miki et al. [17] observed that friction behavior deviates from the Coulomb–Amontons law for polished diamond films. For surfaces with flat plateaus and fine valleys, the friction coefficient becomes speed dependent, influenced by the degree of polishing. They demonstrated experimentally that both the plateau structure resulting from polishing and the sliding speed are critical parameters for friction reduction, governed by the speed-dependent lift and the gap formed by the surface roughness. Ramadoss Radhika et al. [18] found that the wear rate of UNCD coatings increased with applied load, while the friction coefficient remained load independent. The enhanced Raman intensity of UNCD features on the ball scar at higher loads, contrasted with a decreased intensity within the UNCD wear track, confirmed significant transfer layer formation onto the counterface. This wear-rate increase diverged from the observed friction behavior. Predominant friction mechanisms proposed for diamond coatings include graphitization [19,20,21], transfer layer formation [22], and surface passivation [23,24]. The graphitization mechanism involves the thermally induced transformation of diamond (sp^3^) to graphite (sp^2^) under frictional heating, where the sp^2^ phase reduces friction. Consequently, strategies integrating a low surface roughness with effective control of the friction interface represent a crucial approach for enhancing diamond-coating performance.

Diamond-like carbon (DLC) is a metastable material characterized by a mixture of sp^3^ and sp^2^ hybridized carbon bonds, combining advantageous properties of both diamond and graphite [25]. Its exceptional tribological properties—including its high hardness, low friction coefficient, chemical inertness, and substantial thermal conductivity—confer a broad application potential in friction and wear management [26]. Significant progress has been achieved in designing DLC-based superlubricious composite coatings and engineering ultra-low-friction interfaces. For instance, Zhang et al. [27] demonstrated that dopant-induced passivation of dangling bonds or the formation of specific nanostructures (e.g., onion-like graphene) within DLC films can isolate contacting surfaces and promote rolling mechanisms. This reduces interfacial shear forces, thereby enhancing tribological performance [28]. Wang et al. [29] investigated the temperature-dependent (25 °C to 200 °C) friction and structural evolution of DLC films. They identified that temperature modulates the nanostructure of graphene nano-curls, with an optimal match between graphitization and curling occurring at a specific temperature, ultimately governing the attainment of ultra-low friction. However, conventional DLC deposition techniques often face challenges in producing coatings exceeding 20 μm in thickness while maintaining a high adhesion strength. Comparatively, chemical vapor deposition (CVD) diamond coatings exhibit superior mechanical properties, particularly in hardness, adhesion strength, thermal conductivity, and wear resistance, relative to physically vapor deposited (PVD) DLC films. Crucially, the shared carbon allotropy of diamond and DLC results in inherently low interfacial stress and lattice mismatch. This compatibility enables the strategic design of composite coatings featuring a diamond base layer and a DLC top layer. Such architecture leverages the diamond substrate’s robust mechanical properties while utilizing the DLC surface to reduce diamond-coating roughness and exploit its superlubricious potential. Cui et al. [30] synthesized MCD and NCD via hot-filament CVD and subsequently deposited DLC on NCD using femtosecond laser ablation (FLA). The resulting NCD/DLC composite films exhibited reduced friction coefficients, attributed to FLA-induced surface graphitization and enhanced smoothness, minimizing adhered wear debris and interfacial adhesive strength. While laser ablation offers advantages for high-precision localized modifications, it frequently compromises the coating surface quality and exhibits limitations in achieving precise thickness control for diamond-like carbon (DLC) films. Nonetheless, how to achieve the controllable growth of a diamond-like coating on the surface of a diamond coating is very important and of great significance. It facilitates the precise regulation of film thickness, composition, and structure, thereby meeting the stringent requirements of harsh-environment applications. Notably, research on the design and tribological properties of DLC films deposited specifically onto diamond surfaces remains scarce.

Bearing these considerations in mind, this study combines PVD and CVD deposition technologies to fabricate diamond/DLC composite coatings with a controllable thickness on silicon carbide substrates, and systematically evaluates their tribological properties under dry sliding conditions. For the experiment, we selected two typical types of diamond coatings, namely microcrystalline diamond coatings and nanocrystalline diamond coatings, as the research objects. The morphology and structure of the as-deposited coatings and post-tribology wear tracks were comprehensively characterized. Furthermore, the underlying friction mechanisms governing the enhanced performance were elucidated. This study focuses on providing a feasible solution to the problem of severe wear of high-roughness diamond coatings due to insufficient lubrication under dry friction conditions, and providing theoretical support for the exploration of high-performance diamond coatings in the future by investigating the friction mechanism.

## 2. Experimental

### 2.1. Preparation of Diamond Films

**Figure 1:**
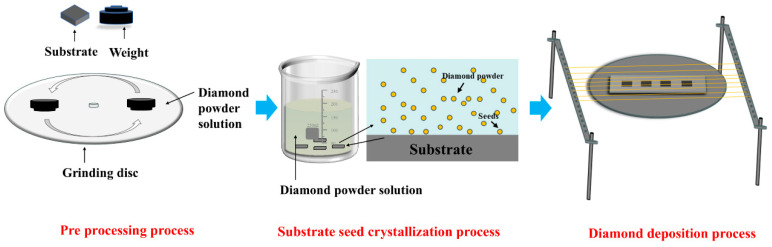
Schematic of the diamond-coating preparation process.

A silicon carbide (SiC) substrate (20 mm × 20 mm × 2 mm, supplied by Fuzhou Infineon Photoelectric Technology Co., Ltd., Fuzhou, China) served as the base for diamond deposition. Microcrystalline diamond (MCD) and ultrananocrystalline diamond (UNCD) films were deposited onto this substrate using hot-filament chemical vapor deposition (HFCVD). Substrate pretreatment: To enhance diamond-substrate adhesion, the deposition surface underwent mechanical roughening. This was performed using a Shenyang Kejing UNIPOL-1502 automatic precision grinder/polisher with a W40 diamond abrasive slurry for 10 min. Subsequently, the substrate was ultrasonically cleaned in anhydrous ethanol. Diamond nucleation seeding was then carried out via ultrasonic treatment in a suspension of nanodiamond particles dispersed in anhydrous ethanol for 30 min. Finally, the substrate was dried using a stream of compressed nitrogen. Deposition process: The pretreated substrate was placed into the HFCVD reactor. Tantalum wires (0.35 mm diameter) were employed as the hot filaments. MCD and UNCD films were synthesized by systematically adjusting key deposition parameters. Detailed process conditions are provided in Table 1. The schematic diagram of the experimental process is shown in Figure 1.

### 2.2. Preparation of Diamond/Diamond-like Carbon (DLC) Composite Coating

The SiC substrate that had been deposited with diamond was cleaned and fixed on the rack inside the chamber of the ion beam equipment. After the chamber was closed, the vacuum treatment was performed. When the vacuum degree in the chamber reached 3 × 10^−5^ Torr, argon was used as the working gas. A bias voltage of −200 V was applied to the substrate. The ion-source current was 0.2 A, and the gas flow rate fluctuated between 30 and 40 sccm to maintain the ion-source operating voltage at 1200 ± 100 V. The surface of the substrate was etched by ionized argon ion, and the process was maintained for 30 min. The ion beam and the bias power supply were turned off, and the gas introduced was replaced by methane/acetylene. DLC was deposited by controlling the gas flow ratio of methane/acetylene. A bias of −100 V was applied to the substrate. The ion-source current was 0.2 A, and the gas flow rate was controlled to fluctuate between 30 sccm and 40 sccm to maintain the ion-source operating voltage at 1200 ± 100 V, and the DLC film with a thickness of about 1 μm was deposited. The flow rate of acetylene was 35 sccm. The growth rate was 800 nm/h, and the temperature of the deposition chamber was 50 °C.

### 2.3. Friction and Wear Test

The tribological properties of the as-fabricated coatings were evaluated using a reciprocating ball-on-flat tribometer under ambient laboratory temperature and dry sliding conditions. A silicon carbide (SiC) ball (radius = 6 mm) served as the counterbody and was loaded normally against the coated substrate with 15 N. Tests employed a 5 mm reciprocating stroke length at an average sliding speed of 30 mm/s for a total duration of 1 h. All tests were repeated three times to establish data validity and experimental consistency.

### 2.4. Characterization

The surface and cross-sectional morphology of the coating were observed and analyzed by cold field scanning electron microscopy and high-resolution transmission electron microscope. The structure of the coating before and after friction was characterized using a laser confocal micro-Raman spectrometer. The cross-sectional area of abrasion marks and paired ceramic balls after friction experiments were observed using a laser confocal microscope. X-ray diffraction (XRD) was used to determine the crystal structure of different diamond coatings. The coating surface roughness was measured using a scanning probe microscope (instrument model: Dimension lcon) manufactured by Bruker Scientific Instruments Hong Kong Ltd. (Hong Kong, China) XPS analysis of elemental bonding states pre- and post-tribological testing was carried out. The calculation formula of coating wear rate, *W*, is as follows:

W=V/FL

Here, the unit is mm^3^/Nm; *V* represents the amount of wear in μm^3^, *F* represents the normal load in N, and *L* represents the total length of the wear scar in m.

## 3. Results and Discussion

### 3.1. Surface Morphology and Structure Analysis of Single-Layer Diamond and Diamond/DLC Composite Coatings

**Figure 2:**
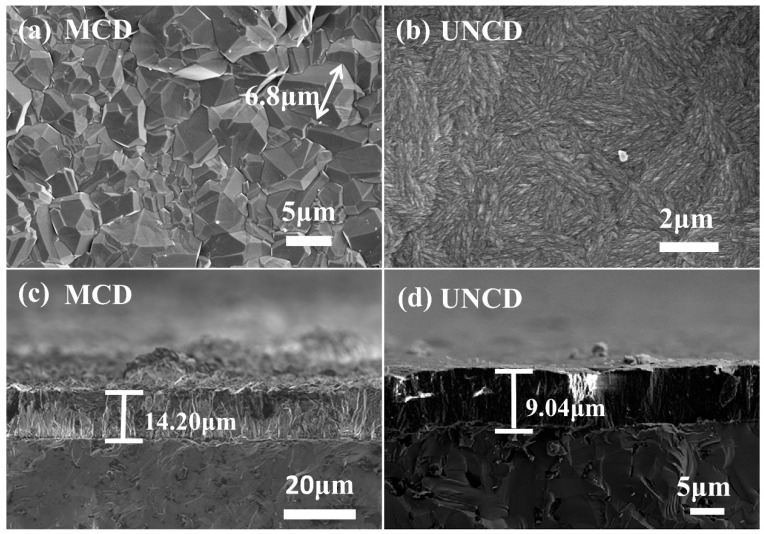
The surface and cross-sectional SEM morphology of (**a**,**c**) MCD coating and (**b**,**d**) UNCD coating.

Figure 2 presents the surface and cross-sectional morphology of the as-deposited MCD and UNCD coatings. MCD coating: The MCD coating exhibits distinct faceted grains indicative of high crystallinity, with sharp edges and well-defined grain boundaries (Figure 2a). Grain sizes range from 3 to 8 μm. The cross-section (Figure 2c) reveals strong adhesion at the MCD/SiC substrate interface. The measured coating thickness is 14.2 μm, corresponding to an average deposition rate of 1.18 μm/h. UNCD coating: Conversely, the UNCD coating displays a significantly refined microstructure, characterized by needle-like or rod-like clusters resulting from its ultra-fine grain size (Figure 2b). This morphology is a recognized characteristic of UNCD films [31]. Cross-sectional analysis (Figure 2d) shows a uniform coating thickness of 9.04 μm, yielding a lower average growth rate of 0.38 μm/h compared to MCD.

**Figure 3:**
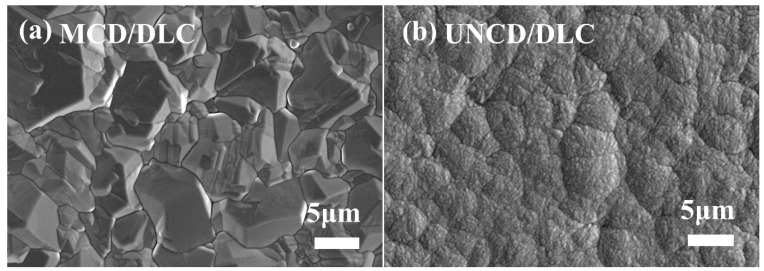
Surface SEM images of (**a**) MCD/DLC and (**b**) UNCD/DLC.

**Figure 4:**
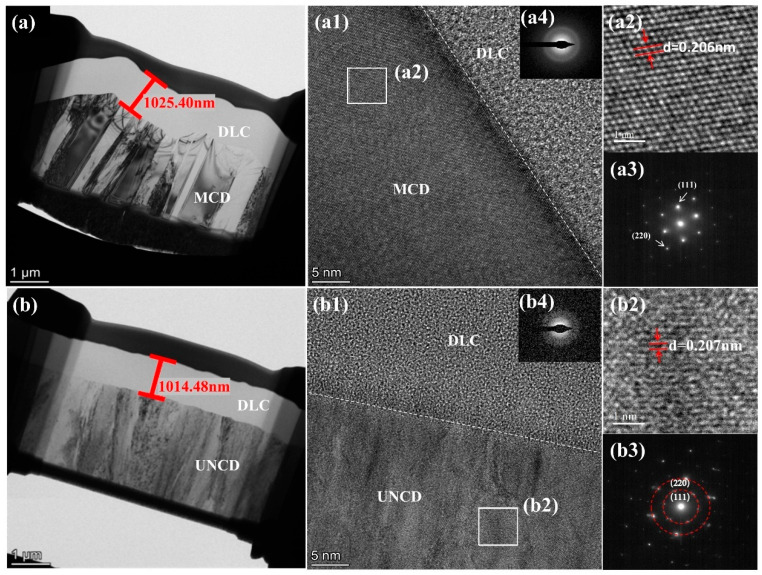
Cross-section TEM images of (**a**) MCD/DLC and (**b**) UNCD/DLC. HRTEM images of the interface of (**a1**) MCD/DLC and (**b1**) UNCD/DLC. TEM images of (**a2**) MCD and (**b2**) UNCD. FT diffraction patterns of (**a3**) MCD, (**b3**) UNCD, and (**a4**,**b4**) DLC.

Figure 3 presents the surface morphologies of the fabricated diamond/DLC composite coatings. MCD/DLC: The MCD/DLC surface morphology (Figure 3a) shows minimal alteration compared to the underlying MCD coating. This retention of features is attributed to the inherently large grain size (3–8 μm) and high surface roughness of the MCD layer, coupled with the relatively thin (~1 μm, confirmed by Figure 4) DLC overlayer, which provides insufficient coverage to significantly modify the topography. UNCD/DLC: In contrast, the DLC deposition markedly transforms the UNCD surface morphology (Figure 3b). While the underlying UNCD coating exhibits characteristic needle-like or rod-shaped grain clusters (as seen in Figure 2b), the conformal deposition of the DLC film encapsulates these ultra-fine nanograins. This encapsulation process results in the formation of irregular spherical agglomerates, effectively masking the original acicular morphology of the UNCD.

Figure 4 presents fractured cross-sectional morphologies and a high-resolution analysis of the diamond/DLC composite coatings. MCD/DLC interface: The cross-section (Figure 4a) reveals a well-bonded interface between the columnar-grained MCD coating and the DLC film. Higher-magnification imaging (Figure 4(a1)) confirms a distinct boundary separating the MCD and the amorphous DLC layer. High-resolution TEM (HRTEM) analysis of the MCD (Figure 4(a2)) displays lattice fringes corresponding to the diamond (111) plane, with a measured lattice spacing of 0.206 nm. The associated Fast Fourier Transform (FFT) pattern (Figure 4(a3)) exhibits sharp diffraction spots, further confirming the high crystallinity and quality of the MCD coating. UNCD/DLC interface: In contrast, the UNCD/DLC interface (Figure 4b,(b1)) appears less distinct. Nevertheless, strong interfacial bonding is observed. This diffuse interfacial region is likely attributed to the presence of non-diamond carbon phases on the original UNCD coating surface prior to DLC deposition. HRTEM of the UNCD (Figure 4(b2)) yields a lattice spacing of 0.207 nm for the (111) plane. The corresponding FFT pattern (Figure 4(b3)) shows well-defined (111) and (220) diamond diffraction rings, confirming that the underlying UNCD retains significant crystallinity and quality, albeit potentially with a higher density of grain boundaries or defects compared to MCD. DLC characterization: FFT patterns acquired from the DLC regions of both composites (Figure 4(a4,b4)) display diffuse rings characteristic of the amorphous structure of the DLC film.

**Figure 5:**
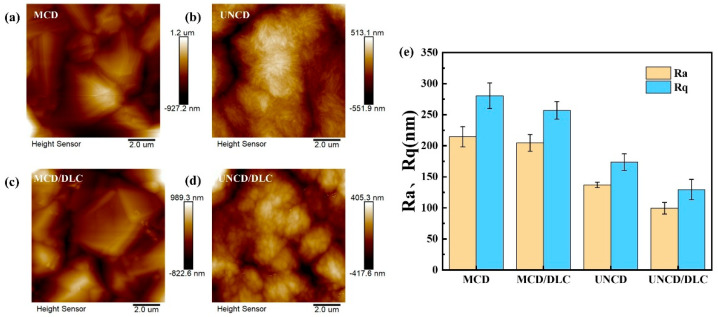
The 2D topographies of (**a**) MCD, (**b**) UNCD, (**c**) MCD/DLC, and (**d**) UNCD/DLC. (**e**) Histogram of Ra and Rq.

Figure 5 presents atomic force microscope (AFM) topography images (10 μm × 10 μm scan area) and surface roughness analysis of the coatings. Morphological evolution: MCD vs. MCD/DLC: The faceted grain edges of the MCD coating (Figure 5a) become notably smoother after DLC deposition (Figure 5c), indicating partial planarization by the carbon film. UNCD vs. UNCD/DLC: The characteristic rod-like clusters of UNCD (Figure 5b) undergo complete morphological transformation, evolving into irregular spherical features upon DLC encapsulation (Figure 5d). Quantitative roughness analysis: The arithmetic mean roughness (Ra) and root-mean-square roughness (Rq) values are as follows: MCD: Ra = 214.5 nm, Rq = 280.5 nm; UNCD: Ra = 137 nm, Rq = 173.5 nm; and MCD/DLC: Ra = 204.5 nm, Rq = 257 nm. UNCD/DLC: Ra = 99.45 nm, Rq = 129.5 nm. Key Observation: DLC deposition reduces surface roughness in all cases, with the magnitude of reduction being strongly correlated to the initial diamond grain size. The UNCD/DLC composite exhibits the most significant roughness decrease (27.4% Ra reduction), while MCD/DLC shows a more modest improvement (4.7% Ra reduction).

**Figure 6:**
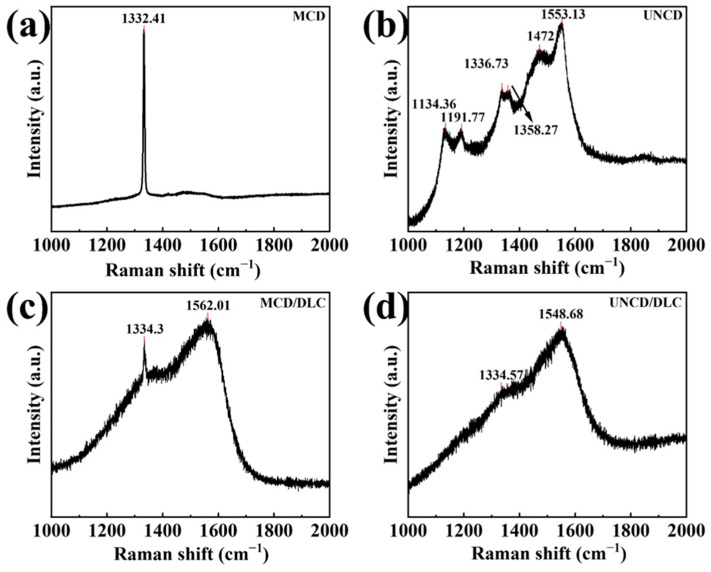
Raman spectra of (**a**) MCD, (**b**) UNCD, (**c**) MCD/DLC, and (**d**) UNCD/DLC.

Raman spectra were further introduced to analyze the structural change in the diamond coating before and after the deposition of the DLC film. As is seen in Figure 6a, a typical diamond characteristic peak near 1332 cm^−1^ can be observed. In addition, the diamond peak in Figure 6a is slender and sharp, indicating that the good quality of the prepared MCD coating. We measured the visible Raman spectra to determine the sp^2^ carbon phase in the UNCD film. As is shown in Figure 6b, it can be seen that the peak at 1336.73 cm^−1^ comes from diamond, while the peaks at 1134.36 cm^−1^ and 1472 cm^−1^ were related to trans-polyacetylene [32]. The peak at 1191.77 cm^−1^ is related to amorphous sp^3^ carbon [33]. Moreover, the peaks at 1553.13 cm^−1^ and 1358.27 cm^−1^ correspond to the G bond and D bond, respectively [34,35,36,37,38]. The Raman spectrum (Figure 6c) of the MCD/DLC coating can be deconvoluted into two contributions: the D peak at 1334.3 cm^−1^ and the G peak at 1562.01 cm^−1^. Similarly, the Raman spectrum (Figure 6d) of the UNCD/DLC coating also shows the D peak at 1334.57 cm^−1^ and the G peak at 1548.68 cm^−1^. Compared with MCD and UNCD, the Raman spectra of MCD/DLC and UNCD/DLC show obvious diamond-like characteristic peaks [39,40], which are caused by the deposition of the DLC film on the surface of diamond coatings. Consequently, it was confirmed that the diamond/DLC composite coatings were prepared successfully on SiC substrates.

**Figure 7:**
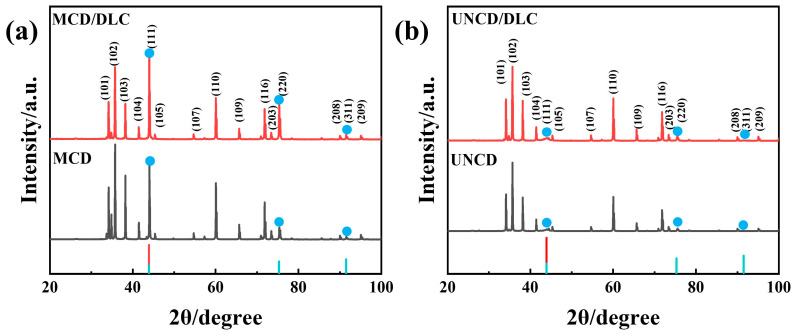
XRD patterns of (**a**) MCD and MCD/DLC, (**b**) UNCD, and UNCD/DLC.

Figure 7 presents XRD patterns of the diamond coatings before and after DLC deposition. The peaks located at 34.182°, 35.728°, 38.234°, 41.503°, 45.286°, 54.623°, 60.153°, 65.806°, 71.967°, 73.593 °, 90.247°, and 95.332° belong to the (101), (102), (103), (104), (105), (107), (110), (109), (116), (203), (208), and (209) crystal planes of the SiC substrate, respectively. Key diffraction peaks observed at 43.9°, 75.3°, and 91.5° correspond to the diamond (111), (220), and (311) planes, respectively [41,42]. Crystallinity differences: The MCD coating exhibits sharp, well-defined (111) and (220) diffraction peaks (Figure 7a). In contrast, the corresponding peaks for the UNCD coating are significantly broadened (Figure 7b). This peak broadening is characteristic of a reduced crystallite size and increased structural disorder, consistent with the transition from microcrystalline to ultrananocrystalline diamond, as reported previously [43,44]. Phase identification: The observed peak positions and relative intensities align with the characteristic diamond diffraction pattern, confirming the successful formation of phase-pure diamond coatings [4]. DLC deposition effect: Crucially, the XRD patterns of the diamond/DLC composite coatings (Figure 7a,b) show no discernible changes compared to their respective underlying diamond coatings. This absence of new diffraction features or peak shifts directly reflects the amorphous nature of the deposited DLC film, which lacks long-range atomic order and therefore produces no sharp Bragg reflections [45,46].

### 3.2. The Tribological Performance of the As-Prepared Coatings

**Figure 8:**
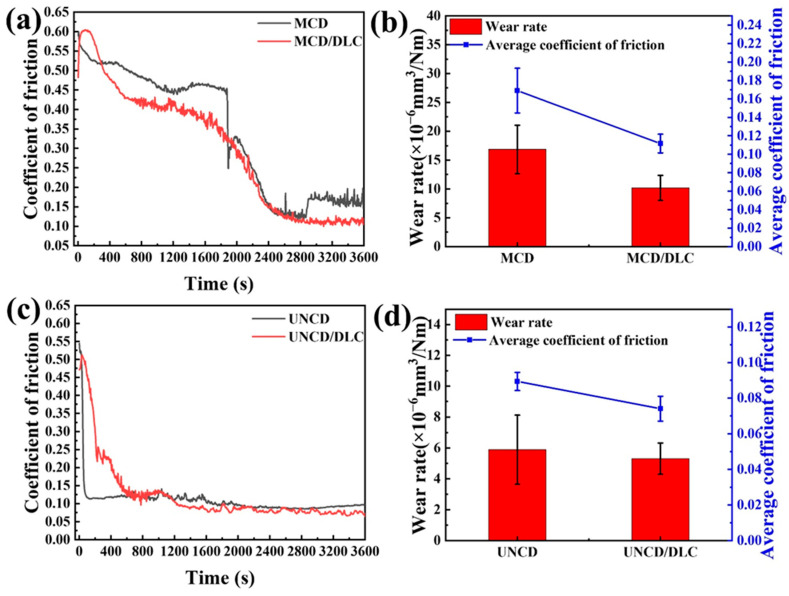
The friction coefficient (COF) curves of (**a**,**c**) diamond coatings and (**a**,**c**) diamond/DLC coatings in dry environment, and corresponding (**b**,**d**) average COF. (**b**,**d**) The average wear rates of diamond coatings and diamond/DLC coatings.

Figure 8 shows the curve evolution of the friction coefficient of different coatings under the same test conditions in a dry environment. The results show that the prepared coatings have a short running-in period at the initial stage of friction process. The friction coefficient of the coating is relatively high at the initial stage of friction, which is also related to the mechanical wear between the coating and the counterpart ball [47]. With the extension of friction time, the mechanical interlocking effect will gradually weaken after the run-in period, and then enter the stable friction period. As is shown in Figure 8a, the running-in period of MCD and MCD/DLC coatings is much longer than that of the other two coatings. Both the large grain size and high surface roughness of MCD lead to an obvious mechanical running-in effect. As is shown in Figure 8a, the friction coefficient of MCD is stable at 0.169 ± 0.024, while the friction coefficient of MCD/DLC is stable at 0.112 ± 0.010, which is 33.73% lower than that of MCD. The wear rate of MCD is 1.684 × 10^−5^ mm^3^/Nm, while the wear rate of MCD/DLC is 1.018 × 10^−5^ mm^3^/Nm, which is 39.55% lower than that of MCD (Figure 8b). Hence, the MCD/DLC composite coating not only reduces the friction coefficient of the MCD coating but also improves its wear resistance. As is seen in Figure 8c, the friction coefficient of UNCD is stable at 0.089 ± 0.005, while the friction coefficient of UNCD/DLC is stable at 0.074 ± 0.007, which is 16.85% lower than that of UNCD. The wear rate of UNCD is 5.88 × 10^−6^ mm^3^/Nm, while the wear rate of UNCD/DLC is 5.31 × 10^−6^ mm^3^/Nm, which is 9.69% lower than that of UNCD (Figure 8d). The results show that the wear resistance of UNCD/DLC is better than UNCD combined with the above tribological performance. According to the above analysis, the introduction of DLC film can effectively improve the friction properties of MCD and UNCD coatings.

**Figure 9:**
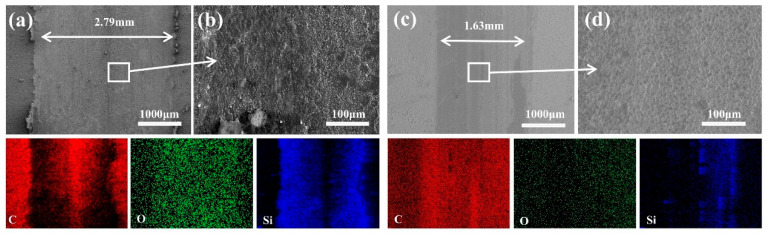
SEM images of (**a**) MCD and (**c**) MCD/DLC. Local SEM-EDS mappings of (**b**) MCD and (**d**) MCD/DLC.

To elucidate the underlying friction mechanisms, the morphology and chemical composition of worn surfaces were systematically characterized. Figure 9 presents comparative wear-scar morphologies and corresponding EDS elemental mappings for both MCD and MCD/DLC coatings. The MCD coating exhibited a scar width of 2.79 mm (Figure 9a), while the MCD/DLC coating demonstrated a significantly reduced width of 1.63 mm (Figure 9c), a decrease of about 41.58%. Beyond scar width reduction, the MCD/DLC composite coating displayed markedly smoother wear-track morphology compared to the grooved surface of monolithic MCD. The MCD coating (Figure 9b) shows pronounced abrasive wear characteristics. In contrast, the MCD/DLC coating (Figure 9d) exhibits substantially diminished abrasive damage and suppressed debris adhesion. This transition in wear behavior directly correlates with the enhanced tribological performance of the composite coating, primarily attributed to the protective function of the DLC overlayer.

**Figure 10:**
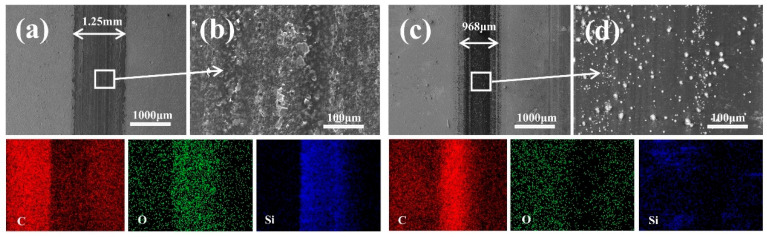
SEM images of (**a**) UNCD and (**c**) UNCD/DLC. Local SEM-EDS mappings of (**b**) UNCD and (**d**) UNCD/DLC.

Figure 10 presents the wear-scar morphologies and corresponding local EDS mappings for both the UNCD and UNCD/DLC coatings. Analysis reveals distinct differences in wear behavior and mechanisms. The wear-scar width for the UNCD coating was measured at 1.25 mm (Figure 10a), while the UNCD/DLC coating exhibited a significantly reduced width of 968 μm (Figure 10c), representing a decrease of about 22.56%. Wear mechanism analysis indicates that the UNCD coating (Figure 10b) displays classical abrasive wear characteristics, evidenced by its grooved morphology. In contrast, the UNCD/DLC coating (Figure 10d) demonstrates a denser tribolayer formation and a significant suppression of abrasive features. This coating also exhibited reduced spallation and minimal surface delamination compared to the MCD coating (Figure 9b), which can be attributed to its lower surface roughness (reduced from 137 nm to 99.5 nm) and reduced interfacial shear stresses.

The EDS results in Figure 10 show a carbon-rich and oxygen-poor phenomenon in the UNCD/DLC abrasion marks, which suggests that the DLC film reduces the occurrence of interfacial oxidation reactions. Whereas, the oxygen-rich region of the diamond-coated friction interface was shown to inhibit the formation of a continuous transfer film and increase the adhesive interaction between the two surfaces [48].

**Figure 11:**
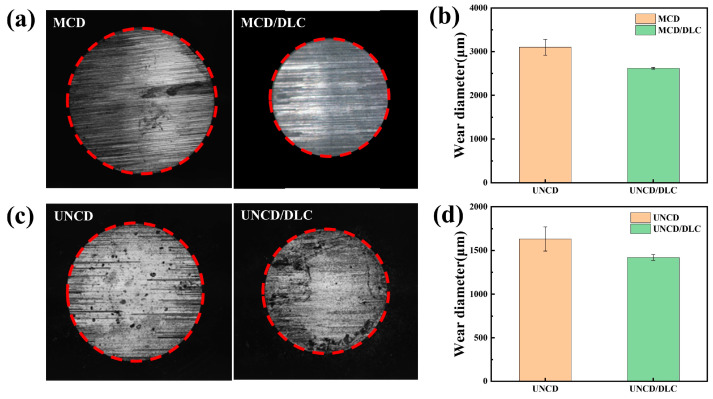
Optical microscope pictures of (**a**,**c**) the wear scars and (**b**,**d**) wear diameters of SiC balls against different coatings in dry environment.

In order to further study the relevant friction mechanism, the optical microscope pictures of the wear scars for SiC ceramic balls sliding across different coatings are shown in Figure 11. It can be seen that the wear-scar sizes on the SiC ceramic balls corresponding to the pristine diamond coatings are higher than those corresponding to the diamond/DLC composite coatings. The surface roughness of the MCD coating is relatively high, and the degree of damage to the SiC balls is significantly higher, and it can be seen that the wear surface of the balls shows obvious furrows and grooves (Figure 11a). However, after the modification of the DLC film, the damage to the surface of the balls has been reduced. The SiC balls ground against the small-size diamond (UNCD) coating had significantly less furrows (Figure 11c), and furthermore, the furrows were almost invisible after the modification of the DLC film. This shows that the DLC film can effectively reduce the furrow effect during the friction process and make the friction interface smoother. From Figure 11b,d, we can see that the average wear diameters of the MCD, MCD/DLC, UNCD, and UNCD/DLC coatings are 3100.25 μm, 2619.70 μm, 1631.75 μm, and 1419.90 μm, respectively. We found that the deposition of DLC can help to decrease the corresponding wear by comparing the counterpart SiC balls of diamond coatings and diamond/DLC composite coatings after friction. This is closely related to the low surface roughness of DLC and the lubrication of the sp^2^ phase interface [49].

**Figure 12:**
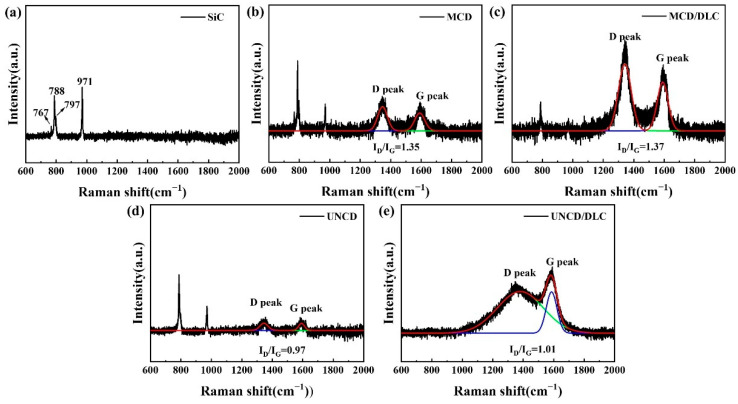
Raman spectra of four coatings corresponding to the wear surface of silicon carbide balls. (**a**) SiC, (**b**) MCD, (**c**) MCD/DLC, (**d**) UNCD, (**e**) UNCD/DLC.

In order to explore the structural changes in the wear surface of silicon carbide balls, we performed Raman characterization of the wear surface. Figure 12a shows the Raman structure of the pristine silicon carbide sphere, and four sub-peaks located at 767 cm^−1^, 788 cm^−1^, 797 cm^−1^, and 971 cm^−1^ can be seen, which are Raman signals from the hexagonal silicon carbide. Unlike the pristine silicon carbide, the four coatings correspond to two extra peaks on the wear surface of the silicon carbide ball: the D peak (1350 cm^−1^) and the G peak (1580 cm^−1^), which suggests that there is a phase transition at the friction interface. The I_D_/I_G_ values are often used as a judgement of the degree of graphitization of the materials. Gaussian fitting of the Raman spectra gives I_D_/I_G_ values of 1.35 (Figure 12b) (The red line: fitting, the green line: D peak, the blue line: G peak), 1.37 (Figure 12c), 0.97 (Figure 12d), and 1.01 (Figure 12e) for the MCD, MCD/DLC, UNCD, and UNCD/DLC coatings, respectively, which suggests that different degrees of graphitization exist at the friction interface of the four coatings. Interestingly, the diamond/diamond-like composite coatings all showed a higher degree of graphitization than the non-composite diamond coatings.

**Figure 13:**
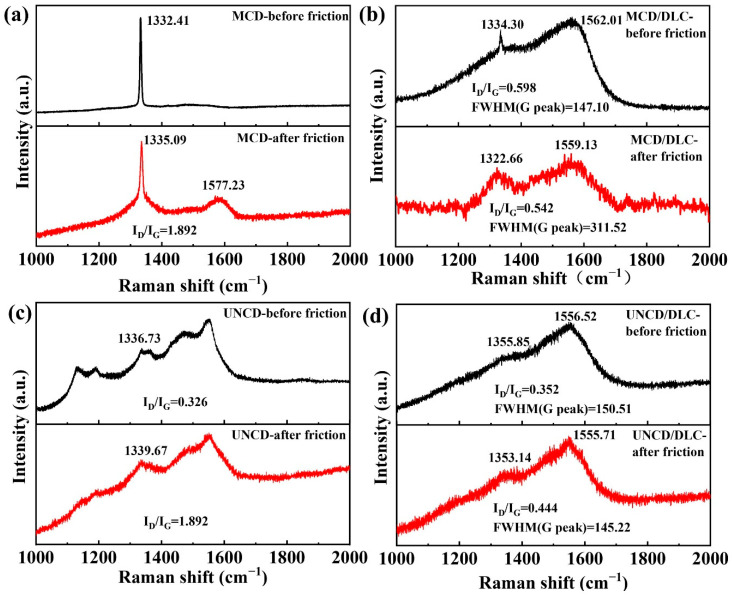
Raman spectra of (**a**) MCD, (**b**) MCD/DLC, (**c**) UNCD, and (**d**) UNCD/DLC before and after friction.

The Raman spectra further reveal the structural changes in as-prepared diamond coatings before and after friction. It can be observed that the diamond peak (1332.41 cm^−1^) shows a right shift after friction (Figure 13a).This may be related to the residual stress caused by non-diamond components and some structural defects [50]. The appearance of the G peak and the increased I_D_/I_G_ ratio further confirmed the friction-induced graphitization transformation mechanism [51,52]. Figure 13b shows the Raman spectra of the MCD/DLC composite coating before and after friction. It can be seen that the peaks at 1334.30 cm^−1^ (D peak) and 1562.01 cm^−1^ (G peak) show left shifts after friction. The shift of the G peak and the increase in the FWHM (full width at half maxima) indicate an augmentation of the sp^2^ phase at the friction interface. These indicate that the graphitization transformation degree of the MCD/DLC composite coating is improved under the action of repeated friction. Similarly, for the UNCD (Figure 13c) and UNCD/DLC coatings (Figure 13d), we also found the graphitization transformation mechanism after friction. Therefore, the friction-induced interfacial graphitization transformation is one of main wear mechanism of a diamond coating. Notably, the introduction of the DLC film will accelerate the graphitization transformation during the friction process, which is closely related to the existence of the sp^2^ phase in the DLC film.

Based on the above analysis, it is reasonable to assume that diamond coatings and diamond/DLC composite coatings have different degrees of oxygen bonding due to the difference in abrasive chips, compared to diamond abrasive chips that are more likely to bond with oxygen to exacerbate the oxygen wear condition during friction. This would result in the diamond coating generating more abrasive debris buildup in the inner pits or on the sides of the wear marks than the diamond/DLC composite coating.

**Figure 14:**
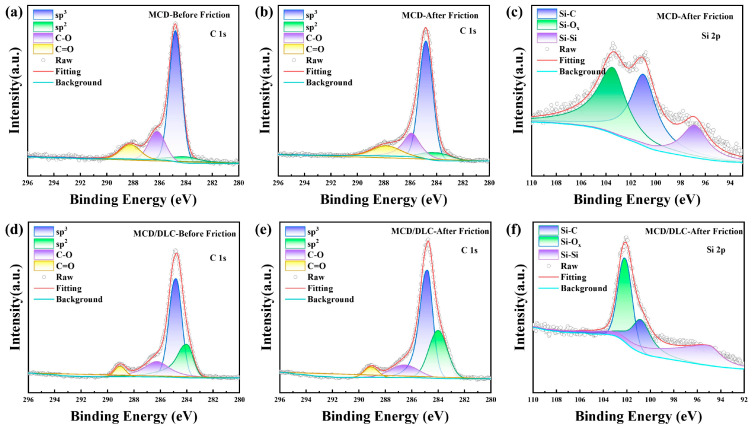
XPS spectra of (**a**–**c**) MCD coating and (**d**–**f**) MCD/DLC composite coating.

**Figure 15:**
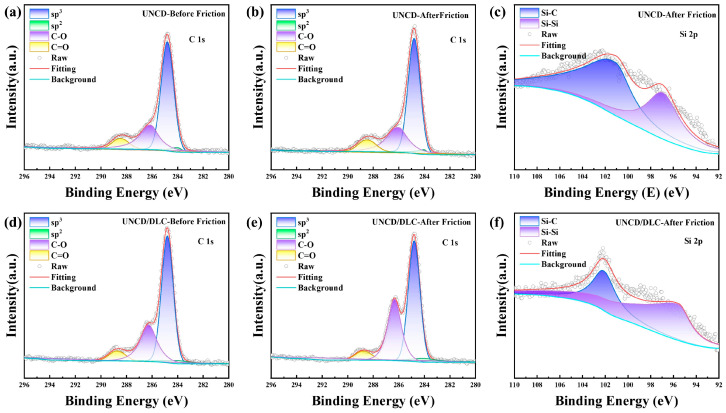
XPS spectra of (**a**–**c**) UNCD coating and (**d**–**f**) UNCD/DLC composite coating.

To further investigate the bonding mechanisms at the friction interface, the coatings were characterized by XPS. Gaussian-fitted spectra are presented in Figure 14 and Figure 15. The parameters of the XPS patterns of C1s and Si2p are shown in Table 2 and Table 3, respectively. These results demonstrate an increase in the sp^2^-phase content for all coatings following tribotesting, indicating that a phase transition occurred at the interface (Figure 14a,b,d,e and Figure 15a,b,d,e). Analysis of the C1s spectra reveals the presence of C-C, C-O, and C=O bonds. The minimal change in these oxygen-containing carbon bonds before and after friction suggests that elemental carbon is not the primary site for oxygen adsorption.

The Si2p spectra acquired post-friction on the large-grained MCD coating show significant detectable oxygen both before and after DLC film compositing (Figure 14c,f). This oxygen bonds with silicon to form silicon–oxygen (Si-O) compounds. Additionally, the spectra confirm the presence of Si-C and Si-Si bonds at the interface. A minor portion of these Si-C and Si-Si bonds originate from the SiC sphere counterbody. Crucially, the Si-O bonds represent new species formed exclusively during friction, demonstrating that the SiC underwent a chemical reaction with airborne oxygen-containing species to produce silicon–oxygen compounds.

In contrast to the MCD coating, detectable Si-O bonds were not present in the UNCD coating both prior to and following DLC film compositing(Figure 15c,f). This suggests that oxidation at the friction interface was less pronounced for both the UNCD coating and the UNCD/DLC composite coating. Notably, the Si2p spectrum of the UNCD/DLC composite coating exhibited a lower SiC content compared to the standalone UNCD coating. This reduction confirms that the combination of the small-grained UNCD coating with the DLC film resulted in a decrease in the amount of generated SiC abrasive debris.

### 3.3. Friction Mechanism of Diamond Coatings with DLC Design in a Dry Environment

**Figure 16:**
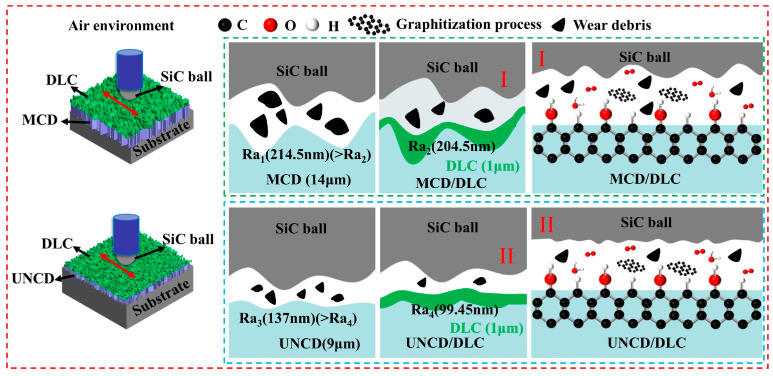
Schematic diagram of wear mechanism of diamond/DLC coatings in air environment.

Based on the previous analysis, the friction mechanism of diamond/DLC composite coatings under dry sliding conditions can be explained as follows: While monolithic diamond coatings primarily exhibit abrasive wear in dry environments [53], the application of a DLC overlayer fundamentally alters the interfacial behavior. The DLC coating significantly reduces the surface roughness of the underlying diamond substrate, leading to a smoother contact interface. This diminished surface topography mitigates ploughing (grooving) effects and consequently reduces wear severity. Crucially, the DLC film, inherently richer in sp^2^-bonded carbon compared to diamond, facilitates friction-induced graphitization. This process occurs more readily and is accelerated within the DLC layer, generating lubricious graphitic phases at the sliding interface. Consequently, the enhanced lubrication mechanism of the diamond/DLC composite coatings is attributed to a synergistic interplay between (1) the formation of a smoother interface minimizing abrasive wear, and (2) the accelerated generation of lubricious graphitic tribolayers. From the above detailed analysis, a possible friction mechanism is concluded in Figure 16.

### 3.4. Discussion

According to the existing literature [54], single diamond coatings exhibit a relatively high coefficient of friction (COF) (approximately 0.1–0.3) and a propensity for stick–slip phenomena under dry, unlubricated sliding conditions, resulting in accelerated wear. While single diamond-like carbon (DLC) coatings demonstrate a lower COF range (0.05–0.2) [55], their amorphous structure renders them susceptible to phase transformation at elevated temperatures (>300 °C) or under high loads, manifesting as increased COF fluctuation and a significant degradation in wear resistance.

This study successfully fabricated MCD/DLC and UNCD/DLC composite coatings. Dry sliding tests revealed that these composite coatings achieved significantly lower COF values of 0.112 and 0.074 accompanied by corresponding wear rates of 1.018 × 10^−5^ mm^3^/Nm and 5.31 × 10^−6^ mm^3^/Nm. These performance metrics represent a substantial improvement over those reported for single diamond coatings [56].

The enhanced tribological performance is attributed to a synergistic effect within the composite architecture: the underlying diamond layer provides superior resistance to abrasive wear, while the surface DLC layer effectively reduces the interfacial COF, stabilizing it within the range of 0.07–0.15. Consequently, under identical dry friction conditions, the composite coatings demonstrate superior performance relative to single diamond coatings, exhibiting a lower COF, reduced wear rates, and markedly enhanced stability.

## 4. Conclusions

Study diamond/DLC composite coatings were designed and fabricated in this study, which was followed by comprehensive comparative analysis of their tribological performance under dry sliding conditions. By introducing a DLC film to modify the diamond coating, the problem of an elevated coefficient of friction and wear rate due to the dry friction of a single diamond with a high degree of roughness is effectively remedied. The tribological properties of the UNCD/DLC composite coatings are the most advantageous, as they have the lowest coefficient of friction as well as the lowest wear rate. Key findings are summarized as follows:

(1) DLC deposition effectively reduces the diamond coating surface roughness, with a reduction in magnitude being inversely proportional to the diamond grain size (27.4% Ra decrease for UNCD vs. 4.7% for MCD).
(2) Diamond/DLC composites significantly enhance tribological performance: MCD/DLC: 33.73% lower friction coefficient and 39.55% reduced wear rate. UNCD/DLC: 16.85% lower friction coefficient and 9.69% reduced wear rate.
(3) The introduction of a DLC coating can reduce wear using smooth interface morphology, and the sp^2^ phase transition caused by friction promotes the interface graphitization to accelerate the lubrication effect.

This study demonstrates a viable surface engineering strategy for enhancing wear resistance in industrial components via diamond-coating modification. However, the composite coatings designed and prepared in this study possessed good dry friction performance only on the basis of the unconsumed DLC film on the diamond surface layer. Based on these shortcomings, future research will focus on multilayer-coating architectures to extend the service life of DLC-modified diamond coatings and improve economic viability.

## Figures and Tables

**Table 1:** Deposition parameters of MCD coating and UNCD coating.

Parameter	MCD	UNCD
CH_4_/H_2_ (Gas flow ratio)/%	3	3
N_2_/H_2_ (Gas flow ratio)/%	0	5
Power/kW	4.4	3.6
Barometric pressure/kPa	2.3	2.0
Height of wire from base/mm	8	10
Deposition time/h	12	24

**Table 2:** The parameters of XPS spectra (C1s).

Sample	Percentage of Individual Peaks/% (Before Friction)			Percentage of Individual Peaks/% (After Friction)		
	sp^2^	sp^3^	C-O_x_	sp^2^	sp^3^	C-O_x_
MCD	8.64	62.98	28.37	8.27	62.96	28.77
MCD/DLC	26.42	50.01	23.58	30.69	52.27	17.05
UNCD	2.92	62.55	34.53	2.84	61.65	35.51
UNCD/DLC	4.30	61.29	34.41	5.23	54.33	40.45

**Table 3:** The parameters of XPS spectra (Si2p).

Sample	Percentage of Individual Peaks/% (Before Friction)		Percentage of Individual Peaks/% (After Friction)		
	Si-C	Si-Si	Si-O_x_	Si-C	Si-Si
MCD	70.28	29.72	41.80	40.23	17.97
MCD/DLC	31.85	68.15	36.50	25.48	38.02
UNCD	42.48	57.52	~	61.93	38.07
UNCD/DLC	59.81	40.19	~	34.39	65.61

## Data Availability

The original contributions presented in this study are included in the article. Further inquiries can be directed to the corresponding author(s).
